# *Gastrodia elata* Blume and *Zanthoxylum schinifolium* Siebold & Zucc Mixed Extract Suppress Platelet Aggregation and Thrombosis

**DOI:** 10.3390/medicina57101128

**Published:** 2021-10-18

**Authors:** Yong-Deok Jeon, Ji-Hyun Lee, Mi-Ran Park, Ji-Ye Lim, Sa-Haeng Kang, Dae-Ki Kim, Young-Mi Lee

**Affiliations:** 1 Department of Korean Pharmacy, Woosuk University, 443 Samnye-ro, Samnye-eup, Wanju-Gun 55338, Jeollabuk-do, Korea; ydjeon1211jh@woosuk.ac.kr; 2 Department of Immunology and Institute of Medical Science, Jeonbuk National University Medical School, 20, Geonji-ro, Deokjin-gu, Jeonju-si 54907, Jeollabuk-do, Korea; jihyunsh1211@naver.com (J.-H.L.); 84juce@naver.com (J.-Y.L.); daekim@jbnu.ac.kr (D.-K.K.); 3 Department of Oriental Pharmacy, College of Pharmacy, Wonkwang University and Wonkwang-Oriental Medicines Research Institute, Iksan 54538, Korea; pmr1021@ran-solution.com (M.-R.P.); rkdtkgod@naver.com (S.-H.K.)

**Keywords:** *Gastrodia elata* blume, *Zanthoxylum schinifolium* Siebold & Zucc, platelet aggregation, thrombosis, anticoagulation, antithrombotic

## Abstract

*Background and objectives:* Blood vessel thrombosis causes blood circulation disorders, leading to various diseases. Currently, various antiplatelet and anticoagulant drugs, such as aspirin, warfarin, heparin, and non-vitamin K antagonist oral anticoagulants (NOACs), are used as the major drugs for the treatment of a wide range of thrombosis. However, these drugs have a side effect of possibly causing internal bleeding due to poor hemostasis when taken for a long period of time. *Materials and Methods:* *Gastrodia elata* Blume (GE) and *Zanthoxylum schinifolium* Siebold & Zucc (ZS) are known to exhibit hemostatic and antiplatelet effects as traditional medicines that have been used for a long time. In this study, we investigated the effect of a mixed extract of GE and ZS (MJGE09) on platelet aggregation and plasma coagulation. *Results:* We found that MJGE09 inhibited collagen-and ADP-induced platelet aggregation *in vitro*. In addition, collagen- and ADP-induced platelet aggregation were also inhibited in a dose-dependent manner on the platelets of mice that were orally administered MJGE09 *ex vivo*. However, compared with aspirin, MJGE09 did not prolong the rat tail vein bleeding time in vivo and did not show a significant effect on the increase in the prothrombin time (PT) and activated partial thromboplastin time (aPTT). *Conclusions:* These results suggest that MJGE09 can be used as a potential anticoagulant with improved antithrombotic efficacy.

## 1. Introduction

Life expectancy is increasing due to the development of modern medical technology, and accordingly, awareness of healthy life is being strengthened. However, the incidence of cardiovascular diseases, such as arteriosclerosis, angina pectoris, hypertension, myocardial infarction, and heart attack, is steadily increasing due to modern westernized eating habits, smoking, drinking, and drug abuse [1]. Cardiovascular disease, which is a major cause of death along with cancer, is caused by blood circulation disorders. This is caused by a thrombus formed by excessive platelet aggregation in the blood vessel or by the narrowing of the blood vessel by the action of substances in the blood vessel [2]. 

Platelets are important for physiological homeostasis; however, when blood vessels narrow, platelets in the blood are squeezed and damaged, and platelets in the blood aggregate to form clots [3]. Excessive clot formation interferes with blood flow and blocks oxygen and nutrients from the arteries, which can lead to clot-related disorders and abnormal blood flow [4]. Therefore, to prevent blood circulation disorders and cardiovascular diseases, it is important to prevent thrombus formation by platelet hyperaggregation. 

Currently, anticoagulants, thrombolytic agents, non-vitamin K antagonist oral anticoagulants (NOACs), and antiplatelet agents, which inhibit platelet aggregation, have been developed and used for the prevention and treatment of thrombotic diseases. Aspirin, known as a representative anticoagulant, has an excellent effect for inhibiting platelet aggregation. However, continuous use can cause serious side effects, such as gastrointestinal disease, skin necrosis, and internal bleeding [5,6]. Therefore, the importance of developing a safer and more effective platelet aggregation inhibitor using various natural ingredients is highlighted.

*Gastrodia elata* Blume (GE), a perennial plant belonging to the orchidaceae, and *Zanthoxylum schinifolium* Siebold & Zucc (ZS), a deciduous shrub belonging to the Rutaceae, are traditional medicinal herbs that have been used in Northeast Asia since ancient times. These are mainly grown in Korea, Japan, and China. GE contains bioactive compounds, such as 4-hydroxybenzyl alcohol, p-hydroxybenzaldehyde (HBAD), vanillyl alcohol, p-hydroxybenzyl alcohol (HBA), gastrodine, p-dihydroxybenzyl sulfoxide, and vanillin [7,8]. To date, GE has been reported to have antidepressant [9], anti-inflammatory [10], anticonvulsant [11], antioxidant [12], neuroprotective [13], and anti-platelet effects [14]. ZS contains bioactive compounds, such as limonene, citronellal, flavonoid compounds, and fatty acids, which are the main aromatic components [15]. Recently, ZS has been reported to have anti-platelet aggregation [16], antioxidant [17], and anti-cancer properties [18].

As described above, various pharmacological effects of GE and ZS are known, and both exhibit antithrombotic effects. If a composite material of GE and ZS is developed, it is thought that a synergistic effect can be expected in the platelet aggregation inhibitory effect. Therefore, in this study, the antiplatelet and antithrombotic efficacy of a mixed extract of GE and ZS (MJGE09) was evaluated through animal experiments.

## 2. Materials and Methods

### 2.1. Materials and Reagents

NADH solution, pyruvate solution, collagen, ADP, sodium pentobarbital, and aspirin were purchased from Sigma-Aldrich (St. Louis, MO, USA). TXB_2_ EIA kit was obtained from Cayman Chemical Company (Ann Arbor, MI, USA).

### 2.2. Preparation of MJGE09

GE and ZS were supplied by MJ Health Foods Co., Ltd. (Muju, Korea). Fresh GE and ZS were first lyophilized and pulverized to a small size. Then, GE was mixed with three hydrolyzing enzymes, 0.2% Termamyl, 0.2% Celluclast, and 0.2% Viscozyme, to improve the absorption rate efficiency of the plant extract. Purified water (10 times, *w*/*v*) was added to the mixture and reacted at 100 MPa and 50 °C for 30 h in a high-pressure decomposition apparatus (TSL-2L; Innoway, Anyang, Korea). Next, the mixture was inactivated at 100 °C for 10 min and centrifuged at 11,000× *g* for 10 min. The supernatant was filtered through the Whatman filter paper and lyophilized. Dried and pulverized ZS was added to 70% ethanol (16 times, *w*/*v*), and hot water extraction was carried out for 2 h at 75 °C. Then, the ethanol extract was filtered and lyophilized. The two extract powders were mixed at a ratio of 1:3 (GE:ZS) for 1 h.

### 2.3. High-Performance Liquid Chromatography (HPLC) Analysis

The HPLC analysis was conducted using an Elite Lachrom HPLC-DAD system equipped with a UV detector (Hitachi HighTechnologies Co., Tokyo, Japan) and a TSK-gel 100 V (4.6 mm × 250 mm, 5 µm; TOSOH, Tokyo, Japan) equipped with a photodiode array detector with a wavelength set at 310 nm. The temperature of the column oven was 25 °C, and the injection volume was 10 μL. The elution was performed at a flow rate of 1.0 mL/min, using acetonitrile (A) and 0.04% trifluoroacetic acid (TFA) (B) as the mobile phase. The solvent gradient was established according to the conditions listed in Table 1. 4-Hydroxybenzyl alcohol is one of the representative GE ingredients, and we confirmed this ingredient, which we expected to play an important role in antithrombotic pharmacological action, through HPLC.

### 2.4. Animals

Male Sprague–Dawley (SD) rats (8 weeks old, weight 250–300 g, *n* = 30) were obtained from Samtako Bio Korea (Osan, Korea). The animals were housed in a controlled environment in a laboratory animal room at a temperature of 22 ± 2 °C, a relative humidity of 50 ± 5%, and a 12-hour light-dark cycle, with freely supply of a standard diet and clean water. After one week of acclimatization, the animals were randomly divided, and the experiment was conducted. All experiments were conducted in accordance with the Guide for the Care and Use of Laboratory Animals Ethics Committee of Wonkwang University (Ethics Committee approval number: WKU17-19; approved: 17 March 2017).

### 2.5. Preparation of Platelets

The rats were anesthetized using ethyl ether. All blood was collected from the abdominal aorta using a tube containing sodium citrate (3.8%, 1:9 *v*/*v*). Platelet–rich plasma (PRP) was obtained from whole blood by centrifugation at 140× *g* for 10 min. Platelet-poor plasma (PPP) was obtained by further centrifugation at 800× *g* for 5 min. Platelets in PRP were adjusted to 4 × 10^8^ platelets/mL using PPP. PRP was used within 2 h of the separation.

### 2.6. Lactate Dehydrogenase (LDH) Release

The degree of intracellular LDH release due to cell membrane damage was used as an indicator of platelet damage. The LDH release assays were performed as previously described [19]. Briefly, PRP was treated with the vehicle or samples, left at 37 °C for 5 min, centrifuged at 10,000× *g* for 1 min, and the supernatant was separated. NADH solution (0.03% b-NAD; Sigma, St. Louis, MO, USA) and pyruvate solution (2.7 mM pyruvic acid; Sigma) were added to the supernatant to measure the decrease in absorbance at 340 nm. The standard of 100% was taken as the time when all the platelets died by treatment with 1% Triton X-100, and the percentage of LDH released from the PRP was converted.

### 2.7. In Vitro Platelet Aggregation Assay

The platelet aggregation assay was performed with reference to the previous paper [20]. Platelet aggregation was induced using collagen (final concentration, 2 μg/mL) or the ADP receptor analogue peptide (final concentration, 5 µM) as agonists. PRP was placed in plastic cuvettes with stir bars, reacted at 37 °C for 10 min, and MJGE09 (0, 4, 20, and 100 μg/mL) or aspirin (0.1 mM) was added and reacted for 5 min. After confirming the baseline for approximately 1 min, a platelet aggregation-promoting substance (collagen or ADP) was added, and the result was measured at 37 °C for up to 10 min, while stirring at 1200 rpm. Platelet aggregation was measured using an aggregometer (Chrono-Log 700-2; Havertown, PA, USA).

### 2.8. Measurement of TXB_2_ Formation

Thromboxane A_2_ (TXA_2_) is unstable and instantly converted to thromboxane B_2_ (TXB_2_). Therefore, the degree of TXA_2_ formation in platelets was confirmed by measuring TXB_2_ using the TXB_2_ EIA kit (Cayman Chemical Co., Ann Arbor, MI, USA). Rat PRP was pre-incubated at 37 °C for 5 min in the presence of MJGE09 (0, 4, 20, and 100 μg/mL) or aspirin (0.1 mM). Next, collagen (2 μg/mL) was added to PRP and incubated at 37 °C for 5 min, followed by the addition of ethylenediaminetetraacetic acid (EDTA) (10 mM) to stop TXA_2_ formation. After centrifugation at 12,000× *g* for 1 min, the supernatant was collected, and the degree of TXB_2_ formation was measured using the protocol provided in the TXB_2_ EIA kit.

### 2.9. Ex Vivo Platelet Aggregation Assay

The platelet aggregation assay was performed with reference to the previous paper [20]. MJGE09 (0, 8, 40, and 200 mg/kg/day) or aspirin (20 mg/kg/day) was orally administered to male SD rats (*n* = 6 per group) at the same time every day for 7 days. The platelet aggregation capacity was measured by separating PRP in the same manner as described above.

### 2.10. In Vivo Bleeding Delay Time Measurement

MJGE09 (0, 8, 40, and 200 mg/kg/day) or aspirin (20 mg/kg/day) was orally administered to SD rats (*n* = 6 per group) for 7 days, and they were anesthetized with sodium pentobarbital (75 mg/kg, ip) one day later. The body temperature of rats was maintained at 37 °C, the tail was cut with a blade of a knife, and the tip of the tail was immersed in a transparent 15 mL tube containing saline to check the blood spread. The time at which the blood stopped spreading and the bleeding completely stopped was measured.

### 2.11. Ex Vivo Prothrombin Time (PT) Measurement

The PT measurement was performed with reference to the previous paper [21]. PPP was prepared as described previously. PPP (50 µL) was incubated at 37 °C for 3 min. Anticoagulant activity was initiated with thromboplastin and monitored for 2 min. When fibrinogen is converted to fibrin, the change in scattered light due to a change in turbidity was measured by irradiating light at 660 nm. The coagulation time was determined as the time required for the change in scattered light to reach 50%.

### 2.12. Ex Vivo Activated Partial Thromboplastin Time (aPTT) Measurement

The aPTT measurement was performed with reference to the previous paper [21]. PPP (50 µL) was incubated at 37 °C for 1 min. Prothrombin reagent (50 µL) was added to PPP, and the mixture was incubated for 3 min. After the addition of 50 µL of 20 mM calcium chloride (CaCl_2_), the reaction was monitored.

### 2.13. Statistical Analysis

Statistical analysis was conducted using the GraphPad Prism v.5.0 software (San Diego, CA, USA). All data values are expressed as the mean ± standard error of the mean (SEM) of at least three independent experiments. After using one-way analysis of variance (ANOVA), the treatment effect was analyzed using the Bonferroni *post hoc* test. * p < 0.05, ** p < 0.01, and *** p < 0.005 values were considered to be statistically significant between groups.

## 3. Results

### 3.1. HPLC Analysis of the MJGE09 Extract

**Figure 1:**
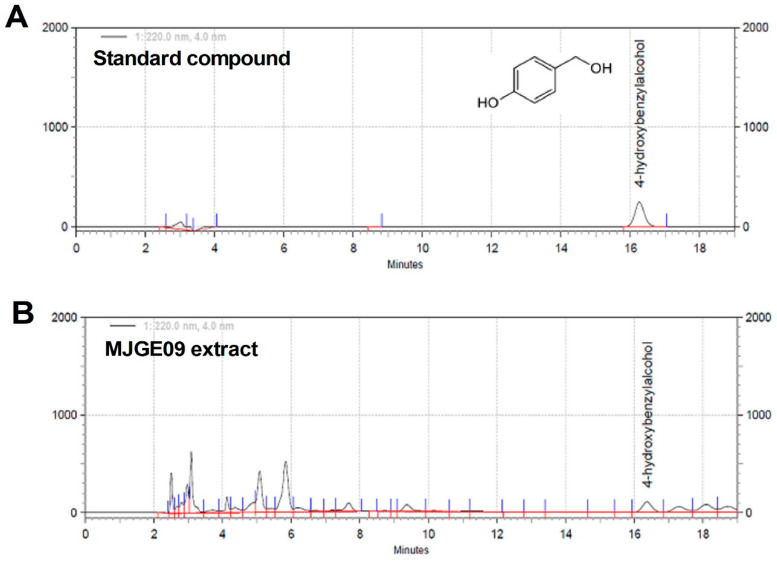
High-performance liquid chromatography (HPLC) analysis of the mixed extract of *Gastrodia elata* Blume (GE) and *Zanthoxylum schinifolium* Siebold & Zucc (ZS) (MJGE09). HPLC chromatograms of the standard compound (**A**) and MJGE09 extract (**B**).

HPLC with diode-array detector (DAD) was used to analyze the content of 4-hydroxybenzyl alcohol, an indicator compound of MJGE09 extract. As shown in Figure 1, the peak of 4-hydroxybenzyl alcohol in the MJGE09 extract was confirmed compared with the peak of the internal standard component. The concentration of 4-hydroxybenzyl alcohol in MJGE09 extract was 182.175 µg/mL (0.182%).

### 3.2. Cytotoxicity Assessment of the MJGE09 Extract

**Figure 2:**
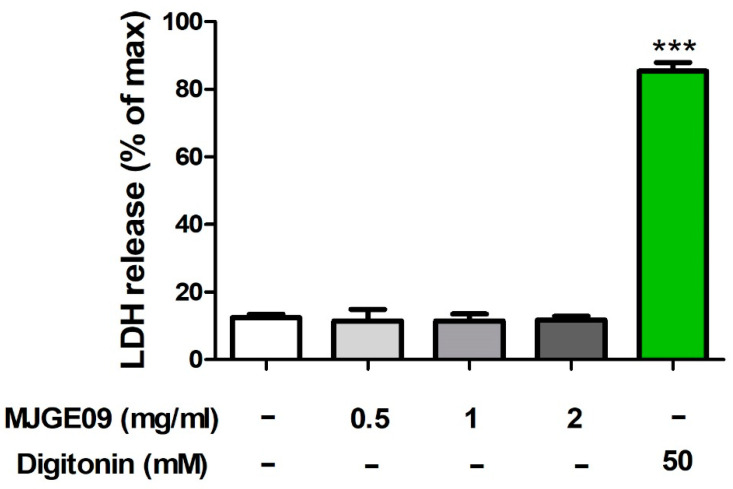
Effects of MJGE09 (0.5, 1, and 2 mg/mL) on cytotoxicity. Cytotoxicity was confirmed by measuring the lactate dehydrogenase (LDH) release in platelets after incubating the rat platelet–rich plasma (PRP) with the vehicle or samples for 5 min. Data represents the mean ± standard error of the mean (S.E.M) (*n* = 6). Statistical significance was set at *** *p* < 0.001 when compared with the control group.

To examine the cytotoxicity of MJGE09 extract, we measured the LDH release in platelets. The mild detergent digitonin was used as a positive control compound, which induces damage to cell membranes. After the PRP was treated with MJGE09 extract (0, 0.5, 1, and 2 mg/mL) or digitonin (50 µM) for 5 min, LDH release was measured. As shown in Figure 2, platelets treated with all concentrations of MJGE09 extracts did not induce LDH release; however, digitonin showed that the platelets were damaged and significantly induced LDH release in the damaged platelets. These results confirmed that MJGE09 extracts were not cytotoxic to rat PRP.

### 3.3. In Vitro Platelet Aggregation Inhibitory Effect of the MJGE09 Extract

**Figure 3:**
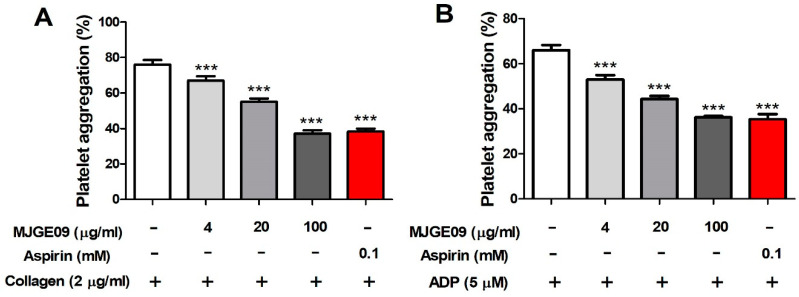
Inhibitory effects of the MJGE09 extract on in vitro platelet aggregation. Platelets were pre-incubated for 5 min with various concentrations of MJGE09 (4, 20, and 100 µg/mL) before being aggregated with 2 µg/mL collagen (**A**) or 5 µM ADP (**B**). Data represent the mean ± S.E.M (*n* = 6). Statistical significance was set at *** *p* < 0.001 when compared with the control group.

To examine the antiplatelet efficacy, MJGE09 extract was pretreated with 8, 40, and 200 µg/mL for 5 min, and platelet aggregation was induced with 2 µg/mL collagen or 5 µM ADP. As shown in Figure 3, MJGE09 extract showed a significant decrease in a concentration-dependent manner. In particular, at a concentration of 100 µg/mL MJGE09, a platelet aggregation inhibitory effect similar to that of aspirin, a positive control, was observed.

### 3.4. Inhibitory Effect of the MJGE09 Extract on TXB_2_ Formation

**Figure 4:**
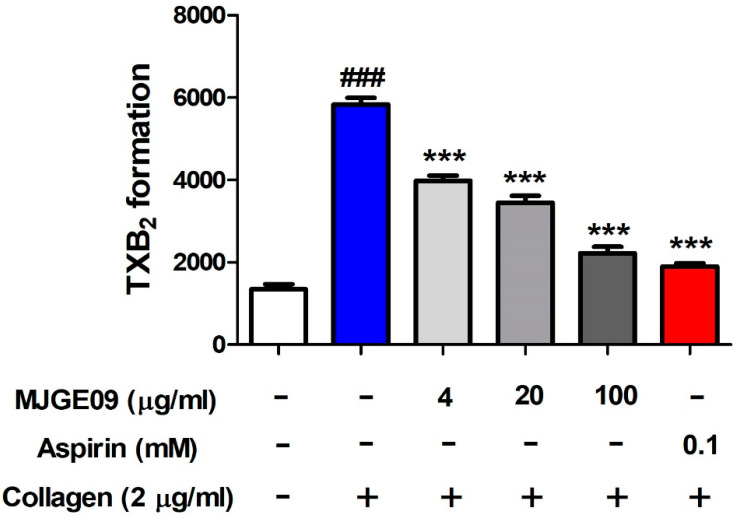
Effects of MJGE09 (4, 20, and 100 µg/mL) on collagen-induced TXB_2_ formation in rat platelets. TXB_2_ formation was determined by the TXB_2_ EIA kit. After pre-incubating the platelet suspensions with saline, MJGE09 (4, 20, and 100 µg/mL) or aspirin (0.1 mM), 2 μg/mL collagen was added. Then, ethylenediaminetetraacetic acid (EDTA) (10 mM) was added to stop TXB_2_ formation. Data represent means ± S.E.M (*n* = 6). Statistical significance was set at ^###^ *p* < 0.001 when compared with the control group, and *** *p* < 0.001 when compared with only the collagen treatment group.

To determine whether the anti-platelet effect of MJGE09 extract is shown by inhibiting TXB_2_ formation, the MJGE09 extract was pre-treated for 5 min, then platelet aggregation ability was observed, and then the degree of collagen-induced TXB_2_ formation was observed. As shown in Figure 4, collagen increased the level of TXB₂ formation to approximately four times (5833.77 ± 161.56 pg/mL) that of the control. However, MJGE09 extract reduced TXB₂ formation in a dose-dependent manner. In particular, TXB_2_ formation by 100 μg/mL of MJGE09 extract (2220.3 ± 154.36 pg/mL) was inhibited to a level similar to 0.1 mM aspirin (1904.5 ± 72.20 pg/mL).

### 3.5. Ex Vivo Platelet Aggregation Inhibitory Effect of the MJGE09 Extract

**Figure 5:**
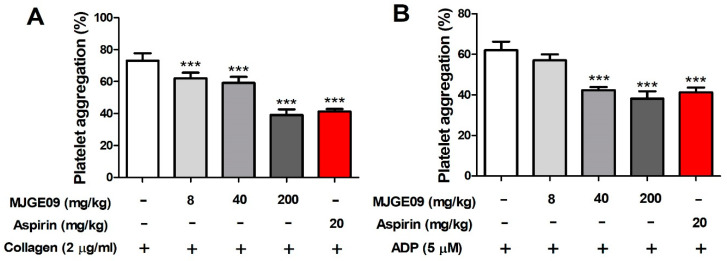
Inhibitory effects of orally administered MJGE09 (8, 40, and 200 mg/mL) on ex vivo platelet aggregation. Platelets from orally administered rats were isolated and aggregation was induced with collagen (2 µg/mL) (**A**) or ADP (5 µM) (**B**). Data represent the mean ± S.E.M (*n* = 6). Statistical significance was set at *** *p* < 0.001 when compared with the control group.

To investigate the antiplatelet efficacy of the MJGE09 extract, the ex vivo platelet aggregation ability was confirmed. As shown in Figure 5, the MJGE09 extract orally administered group (8, 40, and 200 mg/kg) for 7 days inhibited platelet aggregation in a concentration-dependent manner. Similar to the in vitro results, the high concentration 200 mg/kg MJGE09 extract group showed similar inhibitory effects as the positive control, aspirin orally administered group.

### 3.6. In Vivo Bleeding Delay Time Response and Ex Vivo PT and aPTT Assay of the MJGE09 Extract

Current antiplatelet drugs have side effects, such as prolonged bleeding time and impaired blood coagulation. Bleeding time is influenced by the interaction of factors on plasma coagulation and platelet aggregation. To confirm the overall blood clotting delay effect, we first checked the in vivo bleeding time delay response using orally administered MJGE09 extract (8, 40, and 200 mg/kg). Oral administration of the MJGE09 extract extended the bleeding time compared to the control group, but it was confirmed that the effect was lower than that of the positive control aspirin. 

**Figure 6:**
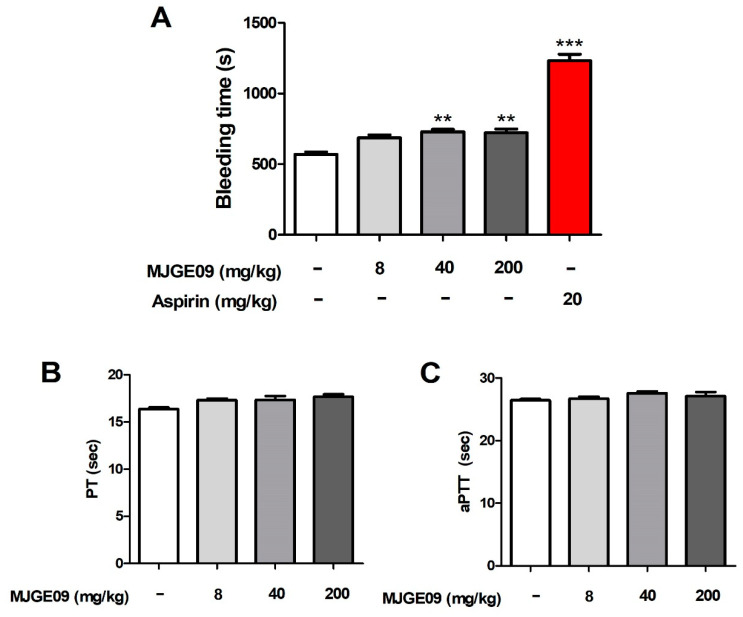
Effects of the MJGE09 extract on mouse tail bleeding times in vivo, and on the prothrombin time (PT) and activated partial thromboplastin time (aPTT) ex vivo. After the rats were orally administered the MJGE09 extract (8, 40, and 200 mg/kg), the tail tip was cut with a knife to measure the bleeding delay time (**A**). PT (**B**) and aPTT (**C**) were determined using a plasma coagulation analyzer. Data represent the mean ± S.E.M (*n* = 6). Statistical significance was set at ** *p* < 0.01, and *** *p* < 0.001 when compared with the control group.

Aspirin 20 mg/kg significantly prolonged the bleeding time compared to the control group. This result confirmed that the MJGE09 extract showed a better effect than aspirin on the bleeding delay reaction, which is a side effect of antiplatelet drugs (Figure 6A). To determine whether MJGE09 affects the plasma clotting time, PT and aPTT were identified. MJGE09 had no effect on ex vivo PT and aPTT at any of the tested doses (Figure 6B,C). Therefore, we can expect that MJGE09 will have fewer side effects on bleeding compared with other antiplatelet drugs.

## 4. Discussion

In our body, various ‘thrombogenic factors’ and ‘thrombotic inhibitors’ are in a dynamic balance, and thus excessive blood clots do not form under normal conditions. However, thrombi are generated by platelets, macrophages, granulocytes, and fibroblasts in blood vessels when the balance of many complex factors involved in thrombus formation and thrombus inhibition is disrupted. Platelet adhesion and aggregation play important roles in the early stages of thrombus formation [22]. The activation and aggregation of platelets acts as a physiological defense mechanism to stop bleeding when blood vessels are damaged; however, in the pathological conditions of arteriosclerosis or damaged blood vessel walls, various cardiovascular diseases, such as myocardial infarction and thromboembolism, can occur [23]. 

In a pathological state, when blood vessels are injured, platelets attach to the damaged blood vessels and are stimulated by various factors, such as collagen, thrombin, and ADP. These activated platelets secrete ADP, serotonin, and TXA_2_, which can act as aggregation amplification factors and cause platelet hyper-aggregation to cause thrombus and arteriosclerosis [24,25]. Therefore, research on the development of antithrombotic agents is being actively conducted to prevent or treat circulatory diseases caused by blood clots.

Aspirin and warfarin are representative antiplatelet drugs developed to date. Aspirin, a cyclooxygenase inhibitor, is an antiplatelet agent that thins blood by inhibiting platelet activation, and warfarin is an antiplatelet agent that exhibits anticoagulant effects by interfering with the synthesis of coagulation factor of vitamin K [5,26]. However, most antiplatelet drugs treat diseases by inhibiting only one of these mechanisms and have the disadvantage of increasing the risk of peptic ulcer and hemorrhagic disease when taken for a long time.

Recently, the preference for a combination drug that blocks various mechanisms of disease rather than a single drug is increasing, and development is being actively carried out. Natural extracts have been reported to have therapeutic and preventive effects in various diseases compared to single ingredient agents and have the advantage of having fewer side effects that are a problem with a single ingredient drug [27]. Therefore, a synergistic effect that further enhances the anti-platelet effect can be expected by combining natural products. In the future, it will be necessary to develop an antiplatelet agent using natural products that have fewer side effects and can block various mechanisms for activating platelets at once.

Previous studies have shown that both GE and ZS have antithrombotic effects [14,17]. Therefore, we prepared an optimal combination to make a more effective antithrombotic agent by combining these natural products. In addition, unlike previous studies, which were only a simple confirmation, the effect of the natural product mixture on the inhibition of thrombus formation by various stimuli was confirmed through various experimental methods.

In this study, we investigated whether MJGE09, a mixture of GE and ZS, inhibited platelet aggregation both in vitro and ex vivo. We first confirmed the absence of MJGE09 cytotoxicity by observing that high concentrations (2 mg/mL) of MJGE09 did not increase LDH release. In addition, MJGE09 inhibited platelet aggregation (%) in a concentration-dependent manner both in vitro and ex vivo. In particular, it was observed that the high concentration of MJGE09 showed similar effects to the positive control, aspirin. To confirm the platelet aggregation inhibition mechanism of MJGE09, the inhibitory effect of TXB_2_ secreted from platelets activated by collagen was investigated. We found that MJGE09 significantly inhibited TXB_2_ in a concentration-dependent manner in activated platelets.

In general, platelet aggregation is a mechanism that stops bleeding, and antiplatelet agents prolong the bleeding time. However, it is known as a side effect of most antiplatelet drugs because it can cause unexpected internal bleeding when overdosed [28]. Therefore, we confirmed the prolongation of bleeding time and anticoagulant effect, and as a result, it was confirmed that MJGE09 did not show significant prolongation of bleeding time compared to aspirin, a positive control. In addition, MJGE09 at all doses tested had no effect on the ex vivo PT and aPTT.

Summarizing the above results, MJGE09 showed significant antiplatelet effects both in vitro and ex vivo. In addition, we confirmed that MJGE09 has an effect on platelet aggregation induced by ADP or collagen. However, this study has limitations because it was an experiment using cells and animals and was not a human study. Additional further studies are needed for applications in humans.

## 5. Conclusions

This research suggests that MJGE09 may exhibit suppressive efficacy against cardiovascular diseases related to platelet hyperactivation, aggregation, and thrombosis. In addition, MJGE09 can compensate for the problems associated with existing antiplatelet drugs by reducing the side effect of internal bleeding caused by prolonged drug usage. However, to develop potent antiplatelet drugs using MJGE09 in the future, follow-up studies are needed to reveal the effective dosage concentrations and additional mechanisms of action of this mixed extract.

## Figures and Tables

**Table 1:** Operating conditions of high-performance liquid chromatography (HPLC).

Parameter	Conditions
Wavelength PDA detector	310 nm
Mobile phase	A: acetonitrile, B: 0.04% TFA A:B = 60:40 (*v*/*v*)
Flow rate	1.0 mL/min
Injection volume	10 μL
Oven temperature	25 °C
Column	TSK-gel 100 V

## Data Availability

Data available on request.

## Abbreviations

aPTT	Activated partial thromboplastin time
GE	*Gastrodia elata* Blume
HPLC	High-performance liquid chromatography
LDH	Lactate dehydrogenase
MJGE09	Mixed extract of GE and ZS
PPP	Platelet-poor plasma
PRP	Platelet–rich plasma
PT	Prothrombin time
SD	Sprague–Dawley
TXA_2_	Thromboxane A_2_
TXB_2_	Thromboxane B_2_
ZS	*Zanthoxylum schinifolium* Siebold & Zucc
